# Assessment of Factors Associated With Non-Compliance to Self-Management Practices in People With Type 2 Diabetes

**DOI:** 10.7759/cureus.18918

**Published:** 2021-10-20

**Authors:** Rahmatullah ., Muhammad Qutubuddin, Rabia Abdul Rahman, Erum Ghafoor, Musarrat Riaz

**Affiliations:** 1 Internal Medicine: Diabetes and Endocrinology, Bolan Medical Complex Hospital (BMCH), Quetta, PAK; 2 Diabetes Education, Baqai Institute of Diabetology and Endocrinology, Baqai Medical University, Karachi, PAK; 3 Dietetics and Education, Baqai Institute of Diabetology and Endocrinology, Baqai Medical University, Karachi, PAK; 4 Diabetes and Endocrinology, Baqai Institute of Diabetology and Endocrinology, Baqai Medical University, Karachi, PAK

**Keywords:** non-compliance to self-management, diabetes education, foot care, self-management, diabetes

## Abstract

Aim and objective

Diabetes mellitus is a chronic metabolic disorder that requires continuous self-management practices. The aim of our study is to assess the factors resulting in non-compliance with self-management practices in people with type 2 diabetes mellitus (T2DM).

Methods

This cross-sectional study was conducted at Baqai Institute of Diabetology and Endocrinology (BIDE), a tertiary care center in Karachi, Pakistan, from March 2019 to May 2019. People with T2DM diagnosed for at least six months were included. A predesigned questionnaire was used to assess various components of self-management such as the use of oral hypoglycemic agents (OHAs) and insulin, self-monitoring of blood glucose (SMBG), physical activity, and daily foot care. Certified diabetes educators conducted interviews on a one-to-one basis. Data were entered and analyzed by using SPSS (version 20; IBM Corp., Armonk, NY).

Results

Better glycated hemoglobin (HbA1c) levels were observed in compliant persons and a statistically significant difference was noted in those who were compliant with insulin use. Good compliance with self-management was observed in people who were given diabetes education previously.

A total of 205 people with T2DM were included in the study, with a mean age of 52.66 ± 11.2 years and a mean duration of diabetes of 8.9 ± 7.5 years. There were 62.9% males and 37.1% females. Oral hypoglycemic agents (OHAs) were prescribed to 62.9% while 33.9 % were on both OHAs and insulin. Non-compliance with the intake of OHAs was 33.3%, insulin injection 21%, SMBG 25.7%, physical activity 69.5%, and foot care practice 34.3%. Various reasons identified for non-compliance included forgetfulness (negligence) (88%), fear of hypoglycemia (10.6%), time constraints (48%), and lack of foot care knowledge (84.8%).

Conclusion

Non-compliance with T2DM self-management is multifactorial and needs continuous reinforcement of structured diabetes education sessions. The study showed that the provision of diabetes education is directly proportional to self-management compliance levels.

## Introduction

Diabetes mellitus is one of the major health issues worldwide. The prevalence of type 2 diabetes (T2DM) is increasing rapidly across the globe. According to the International Diabetes Federation (IDF), 463 million people of ages 20 to 79 years are living with diabetes, which is expected to rise to 700 million by the year 2045 [1]. Sedentary lifestyles and growing obesity among adults and children because of economic development and increasing urbanization with greater consumption of unhealthy foods are some of the contributing factors for this great pandemic [2]. The global prevalence of diabetes in urban areas is 10.8%, in rural areas, it is lower, at 7.2%. Pakistan is now in the top 10 countries for an absolute increase in diabetes prevalence, which has reached 17.1% [3].

A healthy lifestyle is the cornerstone of T2DM self-management. Lifestyle and behavioral modification are the keys to managing T2DM, which includes healthy and balanced diet intake, increased physical activity, and maintenance of healthy body weight [4]. Self-monitoring of blood glucose helps in keeping a balance between medication, activity, and food intake to maintain blood glucose levels close to the normal range. Elevated blood glucose levels increase the risk of both major and minor complications of T2DM and mortality in people with diabetes [5].

Diabetes education is required to educate people to encourage self-management. However, modifying one’s lifestyle is not possible in one session only; it requires constant contact and discussions between the diabetes care team and the person with diabetes, seeking mutually agreed informed decisions and finding ways to implement these in daily life. It is essential that all diabetes care team members, including diabetologists, diabetes care nurses, diabetes educators, registered dietitians, foot care specialists, and pharmacists, should remain on the same page to provide a road map to the person with diabetes for achieving good glycemic control. American Diabetes Association (ADA) recommends the assessment of self-management skills and knowledge of diabetes annually [6].

Compliance is defined as “the extent to which a person’s behavior, such as taking medication, following a diet and/or executing lifestyle changes, corresponds to agreed recommendations from a healthcare provider.” Non-compliance is a major obstacle to the effective delivery of diabetes care. The World Health Organization (WHO) estimated in 2003 that only 50% of people with chronic diseases follow treatment recommendations from developed countries [7]. However, an adherence of 80% or more is required for optimal results [8]. In developing countries, such as Pakistan, the adherence rate is less, and very few people with diabetes achieve good glycemic control, which is indeed a great concern for overall public health. This study highlights the general trend of diabetes management among people with diabetes in Pakistan. Several other studies have been done but the evaluations do not remain the same due to different factors [9-11]. This study aims to highlight the importance of diabetes education in the self-management of diabetes.

## Materials and methods

This cross-sectional study was conducted at Baqai Institute of Diabetology and Endocrinology (BIDE), a tertiary diabetes care center in Karachi, Pakistan. People with T2DM attending the outpatient department (OPD) from March 2019 to May 2019 were included in the study.

Type 1 diabetes, newly diagnosed people with T2DM (less than 6 months), pregnant females, and people with psychotic disorders were excluded. Ethical approval was sought from the institutional review board (IRB).

A predesigned questionnaire was used to evaluate the reasons resulting in non-compliance with self-management practices among the study participants. The questionnaire consisted of 19 questions to assess various factors, such as the use of oral hypoglycemic agents (OHAs) and insulin, self-monitoring of blood glucose (SMBG), physical activity, and daily foot care. Certified diabetes educators conducted interviews on a one-to-one basis after obtaining informed consent from each participant.

Compliance was evaluated in accordance with the frequency of practices by people with diabetes. Participants using insulin/OHAs regularly and performing daily physical activity and daily foot care were considered compliant, whereas those who had erratic self-management practices were considered non-compliant. The study participants who never missed their prescribed OHAs were categorized as having good compliance while those who missed their oral medications once in three months were considered as demonstrating fair compliance. Participants who often skipped or weekly skipped their OHAs were classified as non-compliant [12].

Similarly, people with T2DM who monitored their blood glucose daily were categorized as very good, those who were doing it weekly were considered as exhibiting good compliance, while those who monitored on a monthly basis were considered as fairly compliant.

Statistical analysis

Statistical analyses were conducted by using the Statistical Package for the Social Sciences (SPSS) version 20. Data were presented as numbers (percentage) or mean ± standard deviation. The chi-squared test, Fisher exact test, and student’s t-test were applied as appropriate. P-value <0.05 was considered to be statistically significant. 

## Results

A total of 205 people with T2DM were included in the study, with 62.9% males and 37.07% females having a mean ± SD age of 52.66 ± 11.2. The most frequent reason for skipping medications and not monitoring SMBG was negligence (forgetfulness) while the most frequent reason for not taking care of feet was lack of knowledge and for not exercising was time constraints for people with diabetes (Table 1).

**Table 1:** Reasons for non-compliance with different parameters of DSME DSME: Diabetes Self-Management Education

Patient Characteristics (N=205)		Frequency n (%)	
Age in years	Mean ± SD	52.66 ± 11.2	
Gender	Male	129 (62.9%)	
	Female	76 (37.1%)	
Reason for skipping medicines	Cost	5 (5.6%)	
	Forget	39 (43.8%)	
	Not available	9 (10.1%)	
	Depression	6 (6.7%)	
	Other	30 (33.7%)	
Reason for not monitoring SMBG	Cost	13 (13.3%)	
	Pain	2 (2%)	
	Fear	18 (18.4%)	
	Forget	29 (29.6%)	
	Don’t have time	9 (9.2%)	
	No or faulty glucometer	21 (21.4%)	
	No reason	6 (6.1%)	
Reason for not following foot care advice	Lack of knowledge	67 (84.8%)	
	Forget	10 (12.7%)	
	Lack of time	2 (2.5%)	
Reason for not doing exercise	Lack of time	49 (36.3%)	
	No place of exercise	7 (5.2%)	
	Don’t know how to do	24 (17.8%)	
	Laziness	17 (12.6%)	
	Others (wound, illness, tired)	38 (28.1%)	
Reason for not using prescribed insulin	Cost	2 (1.2%)	
	Fear of injection	3 (1.9%)	
	Complexity	1 (0.6%)	
	Lumps at injection sites	3 (1.9%)	
	Fear of hypoglycemia	17 (10.6%)	
	No effect on glycemic control	6 (3.7%)	
	Not good for health	1 (0.6%)	
	Forget	3 (1.9%)	
	Others	1 (0.6%)	

Table 2 shows the association of non-compliance with glycemic control. Although the majority of the people with diabetes who were non-compliant to any parameter of DSME were found to have bad glycemic control but no statistical significance was found between any parameter of DSME and glycemic control.

**Table 2:** Association of non-compliance with glycemic control

Patient Characteristics (N=205)	HbA1c ≤7% (n=45; 22%)	HbA1c >7% (n=160; 78%)	P-value
Reason for skipping medicines (n=89; 43.4%)			
Cost	2 (9.5%)	3 (4.4%)	0.397
Forgot	9 (42.9%)	30 (44.1%)	
Not available	1 (4.8%)	8 (11.8%)	
Depression	3 (14.3%)	3 (4.4%)	
Other	6 (28.6%)	24 (35.3%)	
Reason of not monitoring SMBG (n=98; 47.8%)			
Cost	5 (26.3%)	8 (10.1%)	0.135
Pain	0 (0%)	2 (2.5%)	
Fear	0 (0%)	18 (22.8%)	
Forget	7 (36.8%)	22 (27.8%)	
Don’t have time	3 (15.8%)	6 (7.6%)	
No or faulty glucometer	3 (15.8%)	18 (22.8%)	
No reason	1 (5.3%)	5 (6.3%)	
Reason of no foot care (n=79; 38.5%)			
Lack of knowledge	16 (80%)	51 (86.4%)	0.388
Forget	4 (20%)	6 (10.2%)	
Don’t have time	0 (0%)	2 (3.4%)	
Reason of not doing exercise (n=135; 65.9%)			
Don’t have time	13 (44.8%)	36 (34%)	0.751
No place of exercise	1 (3.4%)	6 (5.7%)	
Don’t know how to do	5 (17.2%)	19 (17.9%)	
Laziness	2 (6.9%)	15 (14.2%)	
Others (wound, illness, tired)	8 (27.6%)	30 (28.3%)	
Reason of not using insulin (n=161; 78.5%)			
Cost	1 (2.8%)	2 (1.6%)	0.790
Fear on injection	2 (5.6%)	13 (10.4%)	
Complexity	10 (27.8%)	27 (21.6%)	
Lumps at injection sites	1 (2.8%)	2 (1.6%)	
Fear of hypoglycemia	1 (2.8%)	22 (13.8%)	
No effect on glycemic control	2 (5.6%)	17 (13.6%)	
Not good for health	3 (8.3%)	12 (9.6%)	
Forget	0 (0%)	27 (21.6%)	
Other	8 (22.2%)	3 (2.4%)	
Not prescribed	8 (22.2%)	17 (13.6%)	

Table 3 reveals the association of non-compliance with the duration of diabetes mellitus. It shows that among the reasons for not exercising, time constraints, lack of place for exercise, and lack of knowledge of performing exercise was more common in those having a shorter duration of T2DM while laziness and ill-health (foot wound, illness, or being tired) were more common among those who had a prolonged duration of T2DM (> five years).

**Table 3:** Association of non-compliance with the duration of diabetes mellitus

Patient Characteristics (N=205)	Duration of DM		P-value
	≤5 years (n=77; 37.5%)	>5 years (n=128; 62.4%)	
Reason for skipping medicines			
Cost	3(8.6%)	2(3.7%)	0.206
Forgot	16(45.7%)	23(42.6%)	
Not available	1(2.9%)	8(14.8%)	
Depression	1(2.9%)	5(9.3%)	
Other	14(40%)	16(29.6%)	
Reason for not monitoring SMBG			
Cost	5(11.4%)	8(14.8%)	0.548
Pain	1(2.3%)	1(1.9%)	
Fear	6(13.6%)	12(22.2%)	
Forget	15(34.1%)	14(25.9%)	
Don’t have time	2(4.5%)	7(13%)	
No or faulty glucometer	12(27.3%)	9(16.7%)	
No reason	3(6.8%)	3(5.6%)	
Reason for no foot care			
Lack of knowledge	29(90.6%)	38(80.9%)	0.368
Forget	2(6.2%)	8(17%)	
Don’t have time	1(3.1%)	1(2.1%)	
Reason for not doing exercise			
Don’t have time	22(40.7%)	27(33.3%)	0.005
No place of exercise	6(11.1%)	1(1.2%)	
Don’t know how to do	13(24.1%)	11(13.6%)	
Laziness	5(9.3%)	12(14.8%)	
Others (wound, illness, tired)	8(14.8%)	30(37%)	
Reason for not using insulin			
Cost	1(1.4%)	1(1.1%)	0.098
Fear on injection	1(1.4%)	2(2.2%)	
Complexity	1(1.4%)	0(0%)	
Lumps at injection sites	0(0%)	3(3.3%)	
Fear of hypoglycemia	3(4.3%)	14(15.4%)	
No effect on glycemic control	2(2.9%)	4(4.4%)	
Not good for health	1(1.4%)	0(0%)	
Forget	0(0%)	3(3.3%)	
Other	1(1.4%)	0(0%)	
Not prescribed	60(85.7%)	64(70.3%)	

Table 4 shows the association of non-compliance with the status of previous diabetes mellitus education. It shows that among reasons for not using insulin, the cost of insulin, fear of hypoglycemia, and considering insulin not good for health was more common in those people with T2DM who did not receive previous diabetes education while fear of injection, complexity, lumps at the injection site, no effect on glycemic control, and negligence (forgetfulness) were found more in those who had received previous diabetes education (p=0.012).

**Table 4:** Association of non-compliance with the status of previous diabetes mellitus education

Patient Characteristics (N=205)	Previous diabetes education		P-value
	No (n=152; 74.1%)	Yes (n=53; 25.8%)	
Reason for skipping medicines			
Cost	4(5.6%)	1(5.9%)	0.489
Forgot	30(41.7%)	9(52.9%)	
Not available	9(12.5%)	0(0%)	
Depression	4(5.6%)	2(11.8%)	
Other	25(34.7%)	5(29.4%)	
Reason for not monitoring SMBG			
Cost	10(13%)	3(14.3%)	0.607
Pain	1(1.3%)	1(4.8%)	
Fear	15(19.5%)	3(14.3%)	
Forget	24(31.2%)	5(23.8%)	
Don’t have time	7(9.1%)	2(9.5%)	
No or faulty glucometer	17(22.1%)	4(19%)	
No reason	3(3.9%)	3(14.3%)	
Reason for no foot care			
Lack of knowledge	57(85.1%)	10(83.3%)	0.763
Forget	8(11.9%)	2(16.7%)	
Don’t have time	2(3%)	0(0%)	
Reason for not doing exercise			
Don’t have time	40(37%)	9(33.3%)	0.065
No place of exercise	6(5.6%)	1(3.7%)	
Don’t know how to do	23(21.3%)	1(3.7%)	
Laziness	14(13%)	3(11.1%)	
Others (wound, illness, tired)	25(23.1%)	13(48.1%)	
Reason for not using insulin			
Cost	2(1.5%)	0(0%)	0.012
Fear of injection	2(1.5%)	1(3.4%)	
Complexity	0(0%)	1(3.4%)	
Lumps at injection sites	1(0.8%)	2(6.9%)	
Fear of hypoglycemia	15(11.4%)	2(6.9%)	
No effect on glycemic control	3(2.3%)	3(10.3%)	
Not good for health	1(0.8%)	0(0%)	
Forget	2(1.5%)	1(3.4%)	
Other	0(0%)	1(3.4%)	
Not prescribed	106(80.3%)	18(62.1%)	

## Discussion

The present study shows that compliance with self-management practices of people with T2DM is suboptimal. Multiple reasons for non-compliance were found for different components of self-management of diabetes. Although several studies [9-11] have been done to evaluate the reasons for non-adherence, the evaluations do not remain the same due to different factors like level of education, socio-economic conditions, and family structure.

This study's focus was on the following five components, i.e. the use of oral medicines and insulin, physical activity, blood glucose monitoring, and foot care. Findings reveal that the majority of people with diabetes were found non-compliant with physical activity and insulin use. Compliance with OHAs was found to be better than other components of self-management of diabetes. Only 22% of patients had optimum diabetic control (HbA1c ≤7%). In other local studies, as many as 35-75% of diabetic patients have poor control based on their HbA1c [13-14].

More than 70% of the participants took their medications regularly. There were 12.6% of patients who skipped medicines once a week, whereas 9.2% skipped once a month, 12.6% skipped medicines quite often, while 56.3% never skipped. Negligence was found to be the major reason; almost 88% of the participants said they forgot to take medicine. Nearly 43.2% skipped due to different reasons, including attending parties and missing without any valid reason, showing their lack of knowledge about the effect of missing medicines.

A similar study conducted in Iraq in 2018 shows the same main barriers for adherence of participants with anti-diabetic medications, i.e., forgetfulness in seven out of 14 subjects; however, the number of participants was much lower than this particular study [15].

The major reasons for not injecting insulin were fear of hypoglycemia (10.6%), the belief that insulin is not effective in achieving glycemic control (3.7%), forgetfulness (88%), and aichmophobia (1.9%). It was noticed that people often skip the evening dose of insulin because of fear of nocturnal hypoglycemia. Also, sometimes, because of dinner parties, they forget to inject their pre-dinner dose. In comparison, one such study conducted in Saudi Arabia in 2018 showed that among participants with low compliance, the most common factors that contributed to their non-adherence were forgetfulness (40%), fear of hypoglycemia (20%), weight gain (10%), and difficulty with injection (7.1%) [16].

Nearly 64.6% of the study participants were not doing exercise. The most common reason for not exercising was the busy work schedule of the participants. Many participants thought that exercise can only be performed out of home and blamed the lack of public parks as a barrier to exercise. A similar recent study in Delhi, India, showed almost the same level of compliance with physical activity. Similarly, an Iraqi study in 2018 showed illness and busy schedules as barriers to physical activity [17].

Regarding self-monitoring of blood glucose, participants of this study were found much more compliant (50.5%) than participants of a recent Indian study (17.6%) [17]. One of the major reasons for non-compliance with SMBG was found to be anxiety; many of them said, “They are afraid of seeing high values of blood glucose on the meter” so they do not check their blood glucose. Other reasons were forgetfulness, non-availability of glucometers or meter strips, and the cost of monitoring.

Compliance with foot care was also comparatively better. Females were found marginally more compliant than males. A major reason for not doing foot care was a lack of knowledge (84.8%). More than 80% of the non-compliant participants didn’t know that feet should be given special care. Other studies from Pakistan indicate that the prevalence of diabetes foot ulcers ranged from 4.0% to 10.0%. The amputation rate in Pakistan is reported to be high, ranging from 21.0% to 48.0% [18]. Interventions for reducing the risk of foot ulcers include wearing therapeutic footwear and increasing patient education about self-management of diabetes [19].

The provision of diabetes education proved to act as a catalyst in increasing the compliance with self-management among this study participants, resulting in better HbA1c levels and thus decreasing the risk of complications (Table 2, Table 4). Other studies also show that the provision of continuous diabetes education is mandatory to reduce the burden of diabetes and its co-morbidities. Reinforcement is the key to behavioral and lifestyle modification. Keeping glycemic levels within specified limits can only be achieved by the mutual collaboration of people with diabetes and their health care providers, including diabetologists, nurses, dietitians, diabetes educators, pharmacists, and other allied health care professionals.

It is essential to formulate targeted education programs and campaigns to increase awareness and education in the community.

It was also found that motivation and positive attitudes are more effective than providing knowledge alone. The findings of a study in 2011 in Taiwan showed that motivational interviews improved participants significantly in self-management, self-efficacy, quality of life, and HbA1c levels [20]. A recent multinational study revealed gaps in the skills and self-efficacy of people with diabetes and advocated strategies by health care providers for regular educational reinforcement to foster healthy coping with diabetes stress, exercise planning, interpreting blood glucose patterns, and adjusting medications or food to reach the targeted blood glucose levels [21].

## Conclusions

Non-compliance with diabetes self-management is multifactorial and needs continuous reinforcement of structured diabetes education sessions. The study proved that the provision of diabetes education is directly proportional to self-management compliance levels.
